# The Chicago Dental Society

**Published:** 1889-03

**Authors:** 


### Editorial.

### The Chicago Dental Society.

The twenty-fifth anniversary of this body was held in the Grand Pacific Hotel, Chicago, on the 5th, 6th, and 7th of Feb.

The meeting was eminently successful in respect to numbers, to the interest elicited, and the work accomplished. There were in all probably over three hundred in attendance, and in addition there was a large number of students from the various colleges of that city present, so that most of the time of the meeting the large hall in which it was held seemed full.

The reading of papers and discussions began promptly at the opening of the first session, and the time was fully occupied throughout this and every other session of the meeting. The programme had been so arranged that scarcely any mere routine business was introduced, so that it may truly be said, that the time was fully devoted to the reading and discussion of papers which were of great practical interest.

One of the most attractive features of the meeting was, the paper and presentation on the screen, of illustrations of developing tooth-tissues; and also giving illustrations of the presence, location and variety of bacteria in connection with dental caries, by Dr. R. R. Andrews, of Cambridge, Mass. Dr. Andrews is the pioneer in this line of bacteriological work; no one before him has been able to photograph the bacteria found in connection with dental decay. This will doubtless be a great step in the study of this subject, and to Dr. Andrews great credit is due for the success he has attained in this direction.

A portion of two days was devoted to clinics; these were held in the operating rooms of the Chicago Dental College and were under the special supervision of Dr. D. M. Cattell. Many interesting and instructive operations were presented; improvements in bridge work and filling with porcelain inlays were especially attractive features of the clinics. There were so many new points presented in the things exhibited and the operations performed, that the time allotted was too short to afford proper time for sufficient inspection and study. Indeed this is one of the difficulties attending this part of association work everywhere.

Upon the whole the meeting was a very successful one; one that will be productive of much good and will not soon be forgotten by those who were present.

